# Intracellular Overexpression of HIV-1 Nef Impairs Differentiation and Maturation of Monocytic Precursors towards Dendritic Cells

**DOI:** 10.1371/journal.pone.0040179

**Published:** 2012-07-09

**Authors:** Yan Guo, Wen-Wen Xu, Jie Song, Wen Deng, Di-Qiu Liu, Hua-Tang Zhang

**Affiliations:** 1 Key Laboratory of Animal Models and Human Disease Mechanisms of the Chinese Academy of Sciences and Yunnan Province, Kunming Institute of Zoology, Kunming, China; 2 Graduate School of the Chinese Academy of Sciences, Beijing, China; University of Hawaii Cancer Center, United States of America

## Abstract

Nef functions as an immunosuppressive factor critical for HIV-1 replication, survival and development of AIDS following HIV-1 infection. What effects Nef exerts on differentiation and maturation of monocytes towards dendritic cells (DCs) remains greatly controversial. In this study, we used THP-1 (human monocytic leukemia cell line) as monocytic DC precursors to investigate how overexpression of HIV-1 Nef influences the processes of differentiation and maturation of dendritic cells. In striking contrast to negative controls, our results showed that morphological and phenotypical changes (CD11c, CD14, CD40, CD80, CD83, CD86, and HLA-DR) occurred on recombinant THP-1 expressing HIV-1 Nef (short for Nef) upon co-stimulation of GM-CSF/IL-4 or GM-CSF/IL-4/TNF-α/ionomycin. Moreover, CD4, CCR5, and CXCR4 were also down-regulated on Nef. It might be hypothesized that Nef prevents superinfection and signal transduction in HIV-1 infected monocytes. Collectively, our study demonstrates that long-lasting expression of Nef at high levels indeed retards differentiation and maturation of dendritic cells in terms of phenotype and morphology. We are hopeful that potentially, stable expression of intracellular Nef *in vivo* may function as a subtle mode to support long-lasting HIV-1 existence.

## Introduction

Nef, as one of the accessory proteins of HIV-1, has been illustrated in a number of studies on human HIV-1 patients and animal models to function as a major determinant of the pathogenicity of HIV-1 [1], [2]. Monocytes/macrophages are among the principal target cells of HIV-1, and functions of Nef in target cells are generally accepted to account for many aspects of viral pathogenesis [3], [4], [5]. As a regulatory protein expressed earliest and most abundantly in the viral infection cycle, Nef (27kDa) is expressed in the cytoplasm and membrane of infected cells [6], [7], [8]. Although HIV-1 Nef is composed of only 206 amino acids, it is functionally complex, structurally reflected by overlapping effector domains that interact with multiple cellular proteins [9]. The best-characterized function of Nef is its ability to significantly reduce the HIV-1 receptor CD4. Nef removes CD4 from the cell surface by enhancing its endocytosis via recruitment to AP-2 adapter complexes and directing the receptor to lysosomes for degradation [1], [10], [11]. Another well-conserved and defined property of Nef is its ability to down-regulate the cell surface expression of MHC class I (MHC-I) on different cells. In addition, Nef reduces MHC-I expression through the recruitment of AP-1 to the MHC-I cytoplasmic tail to re-route MHC-I from the *trans*-Golgi network to lysosomes [1], [11], [12].

Nef is involved in the impairment of immune responses in several ways [13], [14]. Nef has strong regulatory power to exert suppressing or enhancing effects on cells’ activation and differentiation. HIV-1 impairs the functions of CD4^+^ T cells, CD8^+^T cells, B cells and natural killer cells [15]. However, how HIV-1 Nef influences dendritic cells (DCs) responsible for initiating and modulating immunity remains unclear [16], [17], [18], [19], [20]. Our recent studies have indicated that intracellular over-expression of Rev, Tat, Vif, Vpr and Vpu could induce apoptosis of THP-1 except for Nef.

CD14^+^ monocytes belong to the mononuclear phagocytes system, and initially described as circulating precursors for tissue macrophages. Monocytes have been generally confirmed to act as precursors with the capacity to differentiate into distinct DCs’ subsets under various physiological conditions [21], [22], [23], displaying remarkably diverse functions allowing them to perform multiple defensive functions, from eliminating pathogen by phagocytosis to stimulating antigen-specific T cells’ responses [24]. Nef protects human monocyte-derived macrophages from HIV-1-induced apoptosis, which is one mechanism of forming HIV-1 reservoirs *in vivo* [25]. A large number of studies focus on the well-differentiated DCs interaction with HIV-1 [26], [27], [28]. Moreover, the primary site for HIV-1 entry into body is either blood or mucosal surfaces where circulating monocytes readily encounter HIV-1. Located at the boundary between the inside and outside environments, DCs provide one bridge between innate and adaptive immunity [21]. As sentinels throughout the body, DCs capture, process antigen and undergo maturation, and subsequently migrate to the secondary lymphoid tissues, where they present the processed antigens to naive T cells, and initiate adaptive immune responses. Taken together, DCs derived from either human CD14^+^ monocytes or CD34^+^ hematopoietic progenitor cells (HPCs) must be mature to be capable of strongly stimulating allogeneic T cells’ responses, but immature ones do not as well as precursors. However, effects exerted by Nef on maturation and differentiation of DCs from monocytes are less well-understood, prompting us to examine how HIV-1 Nef affects differentiation of DCs from monocytes. DCs, as professional antigen presenting cells (APCs) arising from either CD34^+^ bone marrow progenitor cells or CD14^+^ monocytes, fulfill critical roles in regulating the immune system [29].

**Figure 1:**
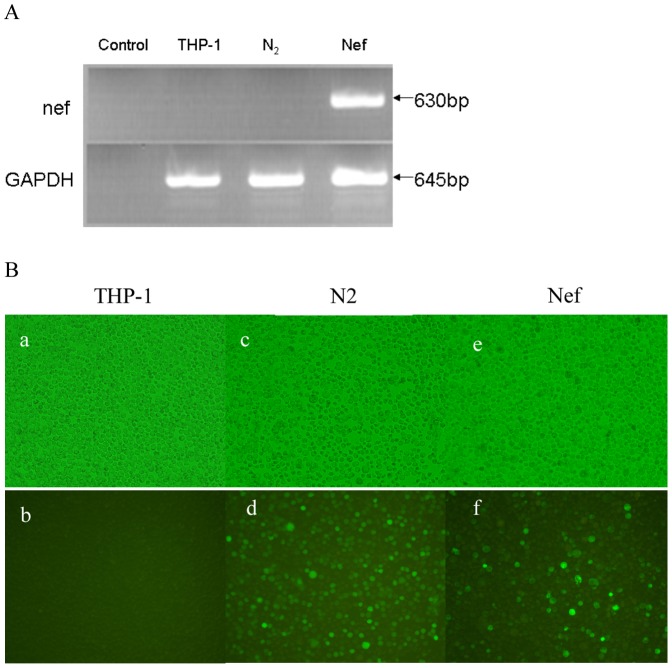
Establishment of stable cell lines Nef and N_2_. The THP-1 cell was transfected with pEGFP-*nef* and pEGFP-N_2_ respectively, giving rise to Nef and N_2_ following the limiting dilution in the presence of 550 µg/ml of G418 within 4 weeks. (A): The visualized PCR product on an ethidium bromide stained 1.2% agarose gel, without any template as controls; (B): a, THP-1 in light background; b, THP-1 in black background; c, EGFP expression by N_2_ in light background; d, EGFP expression by N_2_ in black background; e, Nef-EGFP expression by Nef in light background; f, Nef-EGFP expression by Nef in black background. Microscopy of cells in culture at 400X.magnification.

Human monocytic leukemia cell line THP-1 has been identified as a highly reproducible model for the differentiation of immature DCs (iDCs) and mature DCs (mDCs) in terms of phenotypic, morphologic and functional properties of DCs generated from CD14^+^ monocytes or CD34^+^ HPCs [30], [31] and it has been prove to function as an extremely powerful tool to investigate human DCs’ differentiation and maturation processes. In this paper, we demonstrate for the first time how intracellular over-expression of HIV-1 Nef is able to retard differentiation and maturation of monocytic precursors towards DCs [32], [33].

**Figure 2:**
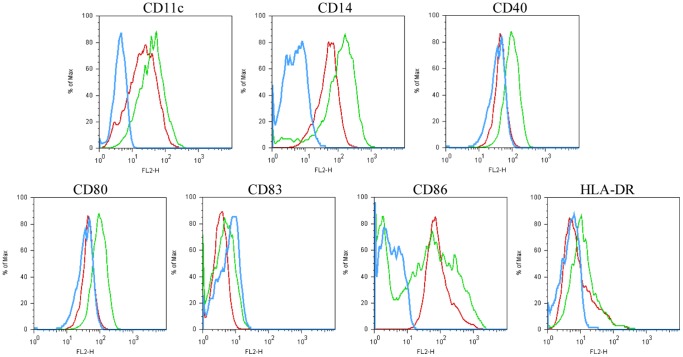
Direct comparisons between phenotype of THP-1, N_2_ and Nef. Surface expressions of CD11c, CD14, CD40,CD80,CD83,CD86,HLA-DR of stable cell lines–Nef and N_2_ (control) within 4 weeks were determined via flow cytometry following establishment. The histograms showed a direct comparison of CD11c,CD14, CD40,CD80,CD83,CD86,HLA-DR levels for Nef *vs* N_2_ and THP-1 Blue line, Nef; green line, N2; red line, THP-1. One representative of three performed experiments was presented.

## Results

### Recombinant THP-1 Stably Expressed Nef

The plasmid pEGFP-N_2_ or pEGFP-*nef* was transfected into THP-1 and then subjected to the first round selection under 100 µg/ml G418 within 4 days and second selection under 550 µg/ml G418 within 4 weeks, finally giving rise to recombinant cell lines pEGFP-N_2_ (short for N_2_) and pEGFP-Nef (short for Nef) and stably expressing HIV-1 Nef. No viable cell was observed in the untransfected cells (short for THP-1) under the same experimental conditions. The gene *nef* was successfully transcribed in recombinant cell line Nef, but not in THP-1 and N_2_ (Fig.1A). Strong green fluorescence was continuously observed in long-lasting drug-resistant cell lines N_2_ and Nef by fluorescence microscopy (Fig.1B). On the same conditions, we tried to construct recombinant THP-1 expressing Vpr, Vpu, Vif, Tat and Rev, but each attempt proved abortive (data not shown).

**Figure 3:**
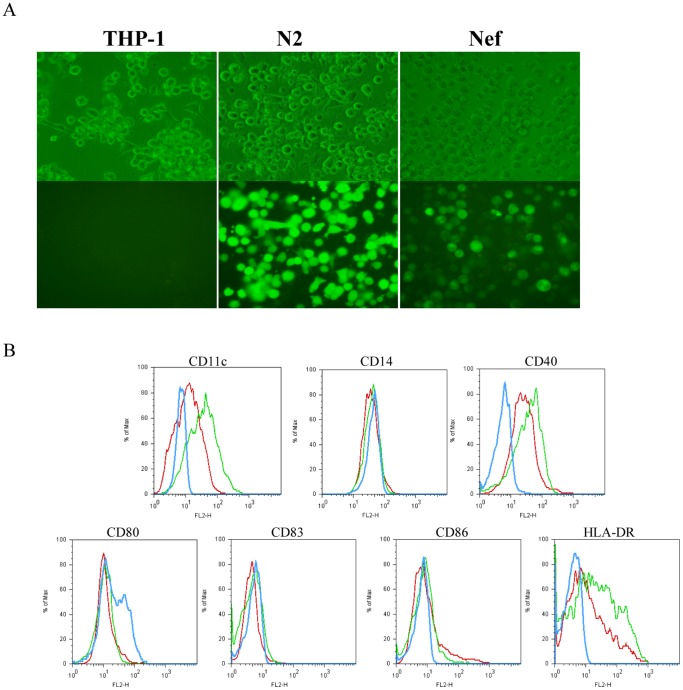
Comparative morphology and phenotype between THP-1, N_2_ and Nef upon co-stimulation with GM-CSF and IL-4 (5-day culture based on “5+0” protocol). (A): morphology of THP-1, N2 and Nef was observed in light background (upper panel) and black one (lower panel) at magnification of 400×; (B): The histograms showed a direct comparison of CD11c,CD14, CD40,CD80,CD83,CD86,HLA-DR levels for Nef *vs* N_2_ and THP-1. Blue line, Nef; green line, N_2_; red line, THP-1. One representative of three performed experiments was presented.

### Nef Induced Different Phenotypes in THP-1 Compared with the Untransfected and N2 Prior to Co-stimulation

We analyzed phenotype (CD11c, CD14, CD40, CD80, CD83, CD86 and HLA-DR) changes to THP-1,N_2_ and Nef prior to stimulation with cytokines. As shown in Fig.2, there was very little difference between THP-1 and N_2_ in the context of these surface markers. It could be hypothesized that GFP hardly interferes with Nef’s biological functions. The levels of CD83 and HLA-DR for Nef *vs* THP-1 and N_2_ had nearly no difference. There was a fine distinction between Nef and THP-1/N_2_ in terms of CD80 or CD40. By contrast, one sharp difference exists between Nef and the latter two upon CD11c, CD14 and CD86. Thus, we initially concluded that Nef expressed in THP-1monocytic precursor for DCs played a certain role.

**Figure 4:**
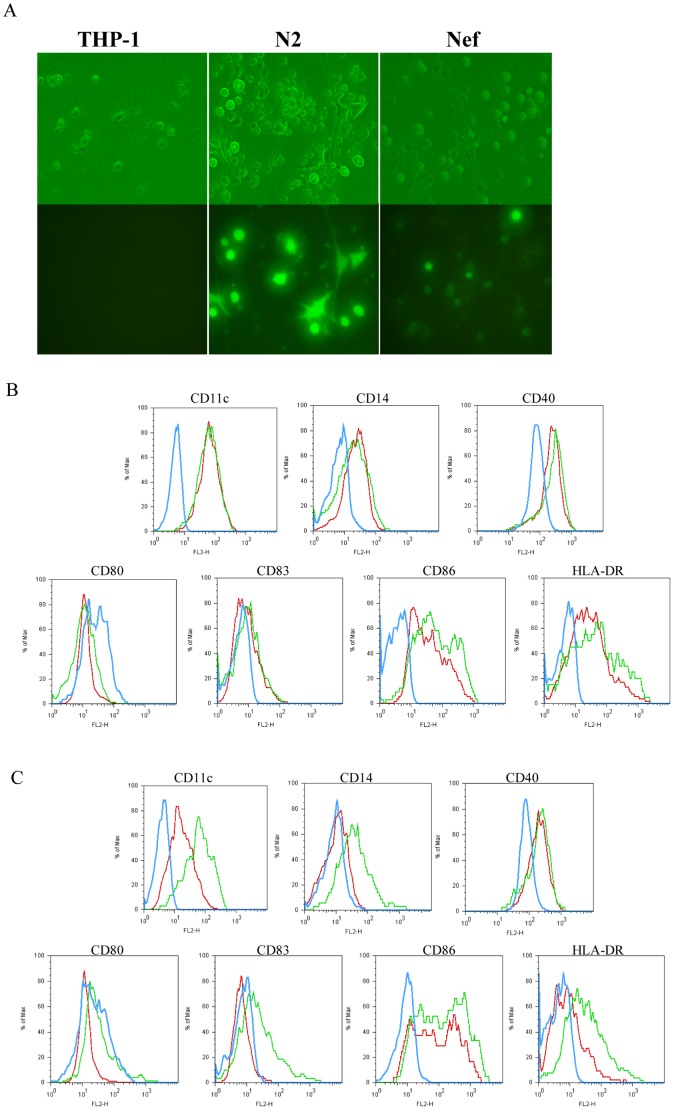
Comparative morphology and phenotype between THP-1, N_2_ and Nef upon co-stimulation with GM-CSF,IL-4,TNFα and ionomycin. (A): morphology of the above cell types was observed in light background (upper panel) and black one (lower panel) at magnification of 400X; (B): The histograms showed a direct comparison of CD11c, CD14, CD40,CD80,CD83,CD86,HLA-DR levels for Nef *vs* N_2_ and THP-1,(7-day culture based on “5+2” protocol ); (C): The histograms showed a direct comparison of above surface markers expression levels for Nef *vs* N_2_ and THP-1 (2-day culture based on “0+2” protocol). One representative of three performed experiments was presented.

**Figure 5:**
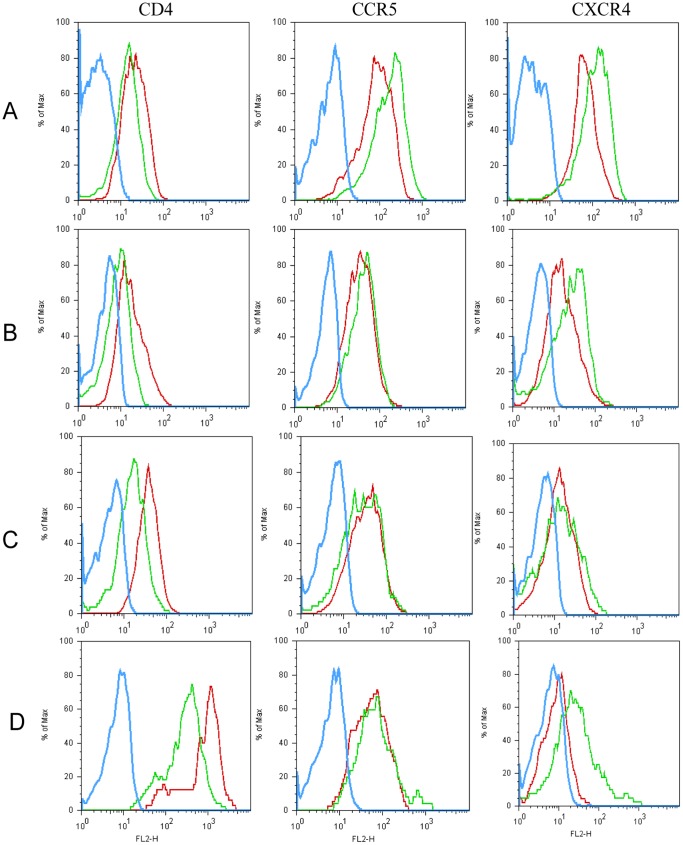
Nef down-regulated CD4, CCR5 and CXCR4 on Nef *vs* THP-1 and N_2_. The histogram shows a direct comparison. Blue line, Nef; green line, N2; red line, THP-1. (A), unstimulated; (B), 5-day culture; (C), 7-day culture;(D): 2-day culture. One representative of three performed experiments was presented.

### Nef Impaired THP-1 Differentiation towards iDCs

On the basis of stable cell lines, rhIL-4 (100 ng = 1500 IU/ml) and rhGM-CSF (100 ng = 1500 IU/ml) were added into three cultures for 5 days (5-day culture), and subsequently flow cytometry and fluorescence microscopy was used to analyze alterations in phenotype and morphology. After 5-day culture in the presence of GM-CSF/IL-4, THP-1 and N_2_ appeared to have characteristic stellate morphology, but Nef did not (Fig.3A). As shown in (Fig.3B), the levels of CD14, CD83, CD86 were supposed to be almost indistinguishable between Nef, N_2_ and THP-1; fine differences existed between Nef and the latter two upon the level of CD80; furthermore, the level of CD11c, CD40 and HLA-DR of Nef were significantly lower than N_2_ and THP-1. From this, Nef impaired THP-1 differentiation towards immature DCs.

### Nef Retarded DCs’ Maturation

TNF-a/ionomycin, as differentiating inducer of mature DCs from THP-1, was supplemented into the above 5-day culture for 2 days (7-day culture). Post co-stimulation with GM-CSF/IL-4/TNF-α/ionomycin, THP-1 and N_2_ displayed typical stellate morphology, but Nef still kept its original shape without any changes (Fig.4A). Nef was significantly lower in the levels of CD11c, CD14, CD40, CD83, CD86 and HLA-DR than THP-1 and N_2_, and appeared slightly higher in the level of CD80 than the other two (Fig.4B), consistent with the results derived from 2-day culture based on “0+2” protocol (Fig.4C). Thus, Nef prevents DCs’ maturation from iDCs or THP-1.

### Nef Down-regulated HIV-1 Receptors on THP-1

Based on unstimulation, GM-CSF/IL-4 stimulation (immature, 5-day culture), GM-CSF/IL-4/TNF-a/ionomycin (mature, 7-day and 2-day culture), Nef displayed significantly lower levels of CD4, CCR5 and CXCR4 just equivalent to background levels of isotypic antibody staining (data not shown) in striking contrast to THP-1and N_2_ (Fig.5).

## Discussion

Numerous studies have identified and adopted THP-1 (human monocytic leukemia cell line) *in vitro* modeling CD14^+^ monocytes or CD34^+^ HPCs that readily differentiate into iDCs and mDCs. THP-1-derived monotypic DCs appear highly pure and displayed the morphologic, phenotypic and functional properties of human CD14^+^ monocytes- or CD34^+^ HPCs-derived DCs, also including endocytotic activity, differentiation associated and IFN-γ enhanceable expression of immunoproteasome subunits and proteasome activator PA28α, and strong T-cell stimulatory capacity [31], [33], [34]. Thus, THP-1 represents a robust and reproducible model to study the effects of Nef on monocyte/DCs biology. Moreover, differentiated THP-1 reflects significant differences between the immature and mature stages in the phenotype *in vitro* [30]. We believe the implications of our results for THP-1 function can be extrapolated to naturally isolated CD14^+^ monocytes, although we clearly cannot rule out the possibility that the effects of Nef expression might be subtly different in natural monocytes.

Over past several years, a large amount of studies focused on Nef’s effects on iDCs developed into mDCs [26], [28], [33], [35], [36]. iDCs have been shown to be immunosuppressive due to their ability to selectively induce the generation and expansion of regulatory T cells (Tregs).Thus, to acquire functional immunostimulating capacity, DCs need to be mature. DCs’ abilities to capture, process and present antigens, activate and modulate T cells, B cells and NK responses, position them at the forefront of therapeutic applications against HIV-1 infection, which has been well documented [19], [37]. However, there is little information available about the effects exerted by Nef on monocytic precursors’ differentiation and maturation towards DCs. Our experimental results revealed that Nef impaired differentiation and maturation from monocytic precursors into DCs in terms of morphological and phenotypic alterations.

On the same conditions, we tried to construct stable cell lines expressing *vif*, *vpu*, *vpr*, *rev* and *tat*, respectively, which ended in failure. These accessory proteins caused THP-1 apoptosis, reflected by Annexin V and PI via flow cytometry (data not shown). This finding also confirms that in advanced stages of HIV-1 infection, a number of DCs diminished with reduced antigen-presenting ability, and per se circulating DCs are rare (<1%) in peripheral blood mononuclear cells (PBMCs) [21], [29]. However, it remains debatable that Nef is associated with a decreased number and dysfunction of DCs during HIV infection. Our results show that expression of *nef* did not result in THP-1 apoptosis. As might be expected, Nef down-regulated the CD4/CCR5/CXCR4 on THP-1, which similarly happened to CD4^+^ T cells in a previous study [10]. Moreover, the cell line Nef completely lost phagocytic capacity in presence of GM-CSF/IL-4 for 5 days in our previous works (unpublished results). These suggest that HIV-1 replicates and latently exists in targets cells for a long time, most likely by restricting superinfection or avoiding apoptosis and necrosis, and then is well-shielded from immune attack in the advanced stages of HIV-1 infection. Intracellular over-expression of Nef has been hypothesized to function as a subtle approach to support HIV-1 replication, survival, latency and escaping immune along with progression of HIV-1 infection. Despite circulating monocytes resistant to productive HIV-1 infection *ex vivo* prior to differentiation into macrophages, HIV-1-infected monocytes have been identified in the peripheral blood of viremic and highly active antiretroviral therapy (HAART)-treated patients, and are considered contributors of viral persistence [38], [39].

Granelli-Piperno *et al* (2004) reported that HIV-1-infected monocytes-derived DCs do not undergo maturation [40]. Nevertheless, another report draws an opposite conclusion, that HIV-1 can induce maturation of monocyte-derived DCs to facilitate subsequent delivery to T cells. This contradiction might result from different stages of HIV infection. Therefore, it remains to be further investigated whether or not Nef plays a decisive role in impairing monocytes’ differentiation and maturation towards mDCs. Interestingly, in this study Nef-expressing THP-1 displayed lower levels of CD14 as compared to N_2_ and THP-1 prior to and post-co-stimulation possibly because THP-1 originated from neoplastically transformed cell lines, and are unable to activate signal transduction pathways involved in up-regulation of CD14 production [41], [42]. CD14 is the first described pattern recognition receptor which also recognizes other pathogen-associated molecular patterns besides lipopolysaccharide (LPS).

In summary, it is reported for the first time that intracellular expression of HIV-1 Nef impairs differentiation and maturation from THP-1 to DCs. Extrapolating our conclusions into CD14^+^ monocytes and CD34^+^ HPCs could also be very worthwhile. Our study offers further insights to help settle the arguments raised by numerous studies on how HIV-1 interferes with immunological functions of DCs. Although we only used a single cell line as a research model throughout the study, it provided the basis for further study in to DCs’ immunological functions in HIV-1 infectious situations. Certainly THP-1 can function as stable and suitable model for HIV-1 infected monocytes/DCs, which need to be further and systematically verified and re-examined using PBMCs as model. We have reasons to speculate that Nef most likely interrupted crosstalk between innate and adaptive immunity during HIV-1 infection via inhibiting monocytes’ differentiation into DCs, especially given the resulting drastic decline in the number of DCs during later stages of HIV-1 infection [43], [44]. Meanwhile, HIV-1 builds up more reservoirs for itself *in vivo*. The different stimuli might confer specific effects on monocyte-derived DCs, reflecting the differentiating plasticity of monocytes. Precisely how Nef interplays with monocytes *in vitro* or *in vivo* under various conditions therefore remains to be explained.

## Materials and Methods

### DNA Procedure

*Nef* gene, derived from SF2 strain of HIV-1, was obtained from the plasmid pHXBnPLAP-IRES-N+(a gift from Prof. Xiaoning Xu, Oxford University, UK) via PCR reaction with specific primers (forward:CGC*GGATCC*ATGGGTGGACAAGTGGTCA, underlined *BamH* Isite; reverse: CGC*AAGCTT*TCAGCAGTCTTTGTAGTACTCCGGAT, underlined *Hind* III site ) performed over 30 cycles at 95°C (1 min), 50°C (1 min), 72°C (1 min), followed by 72°C for 10 min. The PCR product was visualized on an ethidium bromide stained 1.2% agarose gel, and it was recovered and purified with TIANgel Mini Purification Kit (Tiangen, Beijing, China). Subsequently, this pre-treated product with *BamH* I/*Hind* III underwent another round of purification, and were inserted into the corresponding sites of pEGFP-N2 in frame giving rise to pEGFP-Nef and sequenced prior to and after introducing into THP-1.

### RT-PCR

Total RNA extraction and first-strand cDNA synthesis were performed as previously mentioned [45]. The sequences of PCR primer pairs for Nef as above, and for GAPDH as positive control were as follows: forward, 5′-CCATCACCATCTTCCAGGAGCGAG-3′; reverse, 5′CAAAGGTG GAGGAAGTGGGTGTCG-3′. The cDNA template was used in PCR reaction performed over 5 min at 95°C, subsequently 30 cycles at 95°C (1 min), 55°C (1 min), 72°C (1 min), followed by 72°C for 10 min. The PCR product was visualized on an ethidium bromide stained 1.2% agarose gel.

### Construction of Recombinant THP-1

THP-1 was obtained from American Type Culture Collection and cultured in complete RPMI medium (RPMI-1640 with 25 mM HEPES, 2 mM L-glutamine, 100 U/ml penicillin, 100 µg/ml streptomycin and 10% heat-inactivated fetal calf serum). In this study, we established a THP-1 clone expressing a fusion protein composed of Nef and EGFP by introducing pEGFP-Nef plasmid using Cell Line Nucleofector Kit V(Amaxa/Lonza, US) [34] for THP-1 cells according to manufacturer’s recommendations, and cells harboring pEGFP-N2 and without any treatment as controls.

For drug selection of transfected THP-1, the cell culture was first incubated at 37°C for 48 h. The non-adherent cells were decanted and the culture replenished daily with fresh medium containing 100 µg/ml G418 (Sigma) for 4 days. The culture was then replenished every other day with a fresh medium containing 550 µg/ml of G418. Stable drug-resistant cell lines THP-1-EGFP (N2) or THP-1-Nef-EGFP (Nef) clones were established within 4 weeks by limiting dilution in the presence of 550 µg/ml of G418. These recombinant cells were maintained in exponential growth by serial passage in tissue culture flasks in complete RPMI in a humidified incubator at 37°C and 5% CO_2_.

### Detection by Fluorescence Microscopy

After 16 h post-incubation, cultured cells were examined under fluorescence microscopy (Olympus BX51, Tokyo, Japan). Then, the observation of transient EGFP cell was taken once every 12 h till no green fluorescence was detected. Cultured transfected cells were examined once every 12 h till the transgenic cell lines were obtained. Prior to observation, cultures in capped glass culture tubes were first incubated at 4°C for an hour to allow EGFP maturation. Cells were then washed (centrifuged for 10 min at 2,000×g) in ice-cold PBS and fixed in cold 2% formaldehyde. In order to calculate the transfection efficiency, the number of fluorescent THP-1 was counted using a hemocytometer. Where large numbers of cells were present, further dilutions were required.

### Cell Count and Flow Cytometry

Cell counting was performed using a Neubauer counting chamber (Hawksely, UK) with at least 200 cells being counted per sample. Cell viability was assessed using trypan blue dye exclusion.

For surface staining of CD11c, CD14, CD40, CD80, CD83, CD86, HLA-DR, CD4, CCR5 and CXCR4, respectively, cultured cells were washed, suspended at 2×10^5^ cells in 200-µl cold FACS solution and incubated with PE-conjugated mAbs or appropriate isotypic controls for 30 min. All antibodies were obtained from BD Biosciences (San Jose, CA, USA). Cells were then washed twice and resuspended in 300 µl of cold FACS solution. Stained cells were analyzed for two-color immunoﬂuorescence and EGFP expression with a FACSCalibur flow cytometer (BD Immunocytometry Systems, San Jose, CA, USA) assisted by Cell Quest software (BD Pharmingen). A minimum of 10^4^ cells were analyzed for each sample. Cell debris was excluded from the analysis by setting a gate on forward and side scatter to include only cells which displayed the light-scattering properties of viable cells. List mode data were analyzed using FlowJo 8 (Treestar, Ashland, OR, USA).

### Inducing Differentiation of Immature DCs

THP-1, THP-1-EGFP (N_2_) and THP-1-Nef-EGFP (Nef) cells were harvested by centrifugation, resuspended in complete RPMI at a concentration of 2×10^5^ cells/ml, and transferred in a final volume of 18 ml into 6-well culture plates. To induce differentiation, rhIL-4 (100 ng = 1500 IU/ml) and rhGM-CSF (100 ng = 1500 IU/ml) were added. Cells were cultured for 5 days to acquire the properties of immature DCs (5-day culture). Medium exchange was performed every 2 days with fresh cytokine-supplemented medium in a humidified incubator at 37°C and 5% CO_2_, which meant “5+0” protocol [29].

### Inducing Differentiation of Mature DCs

Mature DCs were generated from immature DCs (5-day culture) by addition of rhTNF-a (20 ng/ml  = 2000 IU/ml) and 200 ng/ml ionomycin into the medium for 2 days, which named 7-day culture based on “5+2” protocol [29].

Alternatively, THP-1, N_2_ and Nef cells were harvested by centrifugation, resuspended in serum-free culture medium (without 10% FCS) at a concentration of 2×106 cells/ml, and transferred in a final volume of 18 ml into 6-well culture plates. The following optimized cytokine cocktails were added: rhIL-4 (200 ng/ml  = 3000 IU/ml), rhGM-CSF (100 ng/ml  = 1500 IU/ml), rhTNF-a (20 ng/ml  = 2000 IU/ml), and 200 ng/ml ionomycin. Cells were cultured for 2 days (“0+2” protocol) in a humidified incubator at 37°C and 5% CO_2_ and used for experiments, which named 2-day culture [29].
